# Tobacco Use or Body Mass – Do They Predict Tuberculosis Mortality in Mumbai, India? Results from a Population-Based Cohort Study

**DOI:** 10.1371/journal.pone.0039443

**Published:** 2012-07-27

**Authors:** Mangesh S. Pednekar, Matti Hakama, Prakash C. Gupta

**Affiliations:** 1 Healis, Sekhsaria Institute for Public Health, CBD Belapur, India; 2 Tampere School of Public Health, University of Tampere, Tampere, Finland; McGill University, Canada

## Abstract

Tobacco use and under-nutrition are major public health concerns and tuberculosis is a major cause of morbidity and mortality in India. Using a cohort of 148,173 persons (recruited 1991–1997 and followed-up 1997–2003) the joint effects of tobacco use and BMI on tuberculosis mortality was studied. Tobacco use in any form and low-BMI had joint effect on tuberculosis mortality and the interaction effect was synergistic in men and antagonistic in women. Self-reported tuberculosis was associated with increased risk of tuberculosis mortality. In contrast, no such association was observed for self-reported diabetes persons. The risk pattern remained unchanged even after excluding tuberculosis deaths occurred within 1^st^ two years of follow-up. This study highlights importance of age consideration of individual while excluding early deaths. Around 27% male tuberculosis deaths were attributable to their being underweight and smoker, while 22% male and 37% female deaths were attributable to their being underweight and smokeless tobacco user.

## Introduction

Despite the availability of highly efficacious treatment for decades, tuberculosis remains a major global health problem. In 2010, there were an estimated 8.5–9.2 million cases and 1.2–1.5 million deaths (including deaths from tuberculosis among HIV positive people) [1]. Tuberculosis continues to remain a major burden in developing countries, including India. The WHO estimated that globally the largest burden of tuberculosis deaths in 2010 occurred in the South-East Asia Region, which accounted for 40% of incident cases (3.5 million) and 45% of deaths (0.5 million). However, the estimated incidence rate per 100,000 persons in sub-Saharan Africa was ∼50% higher than that of the South-East Asia Region (276 vs. 193). India ranks first in terms of both number of incident cases (2.3 million) and deaths (0.32 million); accounted for an estimated one quarter of the total burden, globally [1]. The effect of HIV on tuberculosis in sub-Saharan Africa accounted for the massive increase in the incidence of tuberculosis in the last 20 years; however, not much is being reported from India. Additionally, other factors associated with tuberculosis includes; malnutrition, alcohol, immunosuppressive drugs, tobacco smoking and air pollution, and diseases such as diabetes mellitus and silicosis [1*]*–[**3].

The relationship between smoking and tuberculosis has been extensively reviewed [2*]*–[**4] but findings were often based on case control studies [*2*]. In 2007, using Mumbai Cohort Study (MCS) we had reported about the association and concluded that ∼38% of tuberculosis deaths were attributable to smoking, mainly (32%) to bidi smoking [5]. The MCS, based in the largest city in South Asia, was designed specifically to investigate the association between tobacco use and various health outcomes, including tuberculosis. MCS had reported that use of tobacco in any form [*6*], alcohol [*7*] and low body mass index [*8*] {BMI = weight(kg)/height(m)^2^} independently increase the risk of tuberculosis deaths.

Diseases, especially chronic diseases, are almost always caused by multiple risk factors. Estimating the joint effects of multiple risk factors is important because many factors act through complicated pathways [9], [10]. But joint effects were relatively unexplored in epidemiology and population health. First such attempt using MCS was made and reported from India [11], [12]. To continue these efforts now we report the findings of the joint effect of tobacco use and BMI on tuberculosis mortality.

## Methods

### Recruitment

A total of 148,173 persons aged ≥35 years were recruited during 1991–1997 from the main city of Mumbai. House-to-house interviews were conducted face-to-face using a structured questionnaire. Electoral rolls organized by area, with a polling station of 1,000–1,500 persons as the smallest geographical unit, were used as the sampling frame. The electoral rolls provided name, age, sex, and address of all the persons aged ≥18 years. For a selected polling station, all eligible people (aged ≥35 years) listed on respective electoral roll were interviewed by trained field interviewers. The interviews were conducted in local languages (e.g., Marathi, Hindi) but the responses were recorded directly on handheld computers (electronic diaries) in English.

### Data sources

The baseline survey included the measurements of weight (using a bathroom scale that was calibrated to the nearest kilogram) and height (using a measuring tape that was calibrated to the nearest centimeter) and interviewer administration of a structured questionnaire. For this analysis, data regarding potential confounders such as age, sex, education, religion, and mother tongue were abstracted from the baseline survey [13], [14].

### Follow-up

An active house-to-house follow-up was conducted on average 5.5 years after the baseline survey. The field interviewers were provided with the list of names and addresses of all cohort members and were instructed to revisit. If the person was alive and available, a face-to-face re-interview was conducted. If the person was reported as deceased, the date and place of death were recorded with extra questioning and care. Permanent migration from the study area while the subject was alive was considered as withdrawal from the study, and the date of migration was noted. The re-interviews were conducted during 1997–2003. The results of follow-up have been reported earlier [7], [12], [14].

### Cause of death

The deaths recorded during the house-to-house follow-up of MCS were linked with the dataset obtained from the municipal corporation death registers. In Mumbai, almost all deaths are registered and medically certified. For matched deaths, the underlying cause of death was derived from the cause information copied from the corporation death registers and then coded according to the *International Classification of Diseases, Tenth Revision* (ICD-10) guidelines. For 1685 randomly selected matched deaths, an independent death registration verification check was performed and was found to be nearly 100% accurate.

### Statistical analysis

Person-years of follow-up were calculated by using the date of recruitment and the date of endpoint ascertainment (defined as date of expiry, re-interview, or migration) [7], [12], [14]. The response variable tuberculosis death; coded as underlying cause from cause information copied from death registers; was a dichotomous variable (yes/no) and the time to event (or censoring) was regarded as a continuous variable. BMI categories were defined as follows (all units kg/m^2^): extremely thin (BMI<16.0); very thin (BMI 16.0 to <17.0); thin (BMI 17.0 to <18.5); normal (BMI 18.5 to <25.0); overweight (BMI 25.0 to <30.0); and obese (BMI ≥30.0). Details regarding the BMI distribution have been published earlier [14]–[16]. Respondents were broadly classified into those who never used tobacco, used smokeless tobacco only, and those who smoked (may include the use of smokeless tobacco products in addition to smoking). The association between exposure variables (tobacco use and BMI) and outcome (tuberculosis deaths; ICD-10 codes A15–19) were presented as hazard ratios (HR) and 95% confidence intervals (CI) derived from multivariate Cox proportional hazards regression modeling [17] using SPSS 13.0. All Cox models were tested for and met the proportional hazard assumption.

To be consistent and comparable to our earlier reported findings [12], [14]; never tobacco users having BMI 25 to <30 kg/m^2^ was used as the reference group. Gender stratified adjusted HRs and 95% CIs were estimated for the joint effect of tobacco use and BMI on tuberculosis mortality. Analyses were conducted to study the multiplicative (vs. additive) effect of tobacco use and BMI on tuberculosis mortality. Expected HRs for multiplicative model were calculated by multiplying individual HRs for the various categories of tobacco use across BMI and for additive model by adding individual HRs and then subtracting 1 from total. Observed HRs higher than the calculated expected HRs indicated synergistic interaction, in contrast, if it was lower indicated antagonistic interaction.

The population attributable risk was derived by applying the adjusted HRs to a modified version of the Rockhill el al [18] formula **∑pd_i_{(HR_i_-1)/HR_i_}**, where ‘pd_i_’ represents the proportion of the total deaths in the population arising from the i^th^ exposure category and HR_i_ is the (adjusted) HR for the i^th^ exposure category (relative to the reference or unexposed stratum).

*Ethics Statement:* During early 1990 there was no institutional review board in function at the Tata Institute of Fundamental Research where the study initiated. Therefore, the baseline study design, protocol, consent procedures and questionnaires were evolved in collaboration with scientists at the International Agency for Research on Cancer, WHO, and University of Oxford. International guidelines regarding the ethical treatment of human subjects were scrupulously followed.

When the project shifted to Healis – Sekhsaria Institute for Public Health (Healis) in 2005, the study was presented to the independent institute review board of Healis (Healis-IRB). This Healis-IRB (Epidemiological) was formed as per the guidelines provided by the Indian Council of Medical Research (which confirmed to the Helsinki declaration and to local legislation) and was registered with the U. S. Department of Health and Human Services (HHS), NIH, USA with a Federal Wide Assurance. The Healis-IRB approved the informed verbal consent procedure used in the study and permitted data analysis to continue.

## Results

Study sample of 148,173 persons (Table 1) were largely middle-aged (median age, 50 years; interquartile range, 42–59). Women were on an average 4.55 years younger but almost three times more illiterate than men. Around 40% women never used tobacco and 51% had normal BMI, among men these proportions were 30% and 62% respectively. Of the total observed tuberculosis deaths, ∼30% had taken place before the age 50 years. The information on self-reported diseases was available for ∼90,000 persons; out of whom ∼3% reported history of tuberculosis and diabetes at the baseline survey.

**Table 1:** Summary of Descriptive Data by gender, MCS, India.

	Women			Men		
	Overall (n = 59,515)	TB Deaths		Overall (n = 88,658)	TB Deaths	
Variables		All (n = 174)	Excluding 1^st^ 2 yr (n = 126)		All (n = 710)	Excluding 1^st^ 2 yr (n = 469)
*Age*						
35–39	14782	16	13	8377	57	43
40–44	10056	25	20	7043	46	27
45–49	9137	21	18	23706	143	99
50–54	7914	30	24	14316	98	69
55–59	5872	16	12	10772	82	51
60–64	5474	32	22	9805	107	68
65–69	3030	14	9	6740	77	55
70 & up	3250	20	8	7899	100	57
*BMI categories*						
Extremely thin	3420	53	35	3809	245	145
Very thin	2573	21	17	3500	87	59
Thin	5425	23	17	8125	121	77
Normal	30567	63	45	55312	226	162
Overweight	13416	11	9	15547	26	22
Obese	4114	3	3	2365	5	4
*Education*						
Illiterate	26959	109	74	15091	140	93
Primary School	20850	46	36	33549	338	223
Middle School	8196	15	14	26075	162	110
Secondary School	2536	2	1	8308	50	28
College	974	2	1	5635	20	15
*Religion*						
Hindu	48817	139	98	68122	563	371
Muslim	3959	8	7	14224	56	38
Buddhist	4529	23	18	3728	75	52
Christian	1874	3	2	2209	14	7
Others	336	1	1	375	2	1
*Mother Tongue*						
Marathi	44808	151	112	49208	507	333
Hindi	4114	5	5	14989	39	31
Guajarati	3292	8	2	8521	85	60
Urdu	2232	6	5	7863	37	25
South languages	4780	4	2	7899	41	19
Others	289	0	0	178	1	1
*Tobacco use*						
Never – user	23960	46	31	26682	123	84
Smokeless	35290	123	92	34120	279	189
Smoker§	265	5	3	27856	308	196
*Bidi* #	249	5	3	16233	229	144
*Cigarette only*	16	-	-	11623	79	52
*Self-reported diseases*						
No disease	28938	58		59516	393	
TB	60	2		557	35	
Diabetes	356	1		1618	10	

Abbreviation: MCS, mumbai cohort study; TB, tuberculosis; yr, year; BMI, body mass index  =  weight (kg)/height (m)^2^.

§ may include smokers plus mixed (smoking and smokeless) users.

# may include bidi plus cigarette smokers.

**Figure 1:**
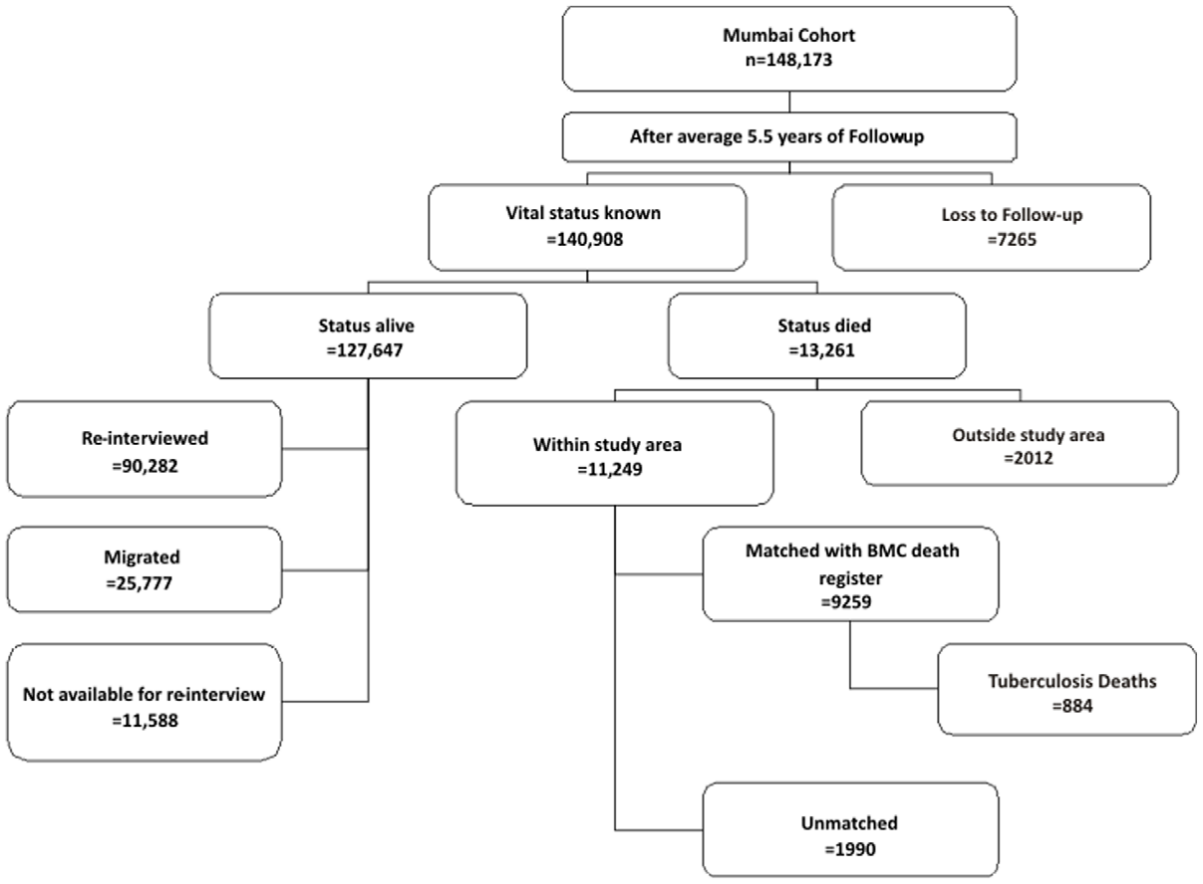
Flow diagram of house-to-house follow-up of the study participants, Mumbai Cohort Study, India.

After an average of 5.5 years of follow-up, 7265 persons could not be traced (Figure 1); the common reason was the demolition of their residential building (6452 persons). Among the remaining 140,908 persons; 13,261 died while 127,647 were alive (of which 25,777 subjects had migrated outside the study area) at the end of the follow-up.

In table 2, we present person years, crude and adjusted (for age, education, religion and mother tongue) HRs along with 95% CIs for various categories of tobacco use across BMI. When compared with reference group, an increased risk of tuberculosis deaths was observed for smokers (men only) and smokeless tobacco users (men and women) across low-BMI. Few women reported smoking; they were excluded from the multivariate analyses. Exclusion of persons who had died in the first two years of follow up yielded reduction in risk estimate but remained statistically significant. The largest such reduction was observed among the extremely thin (<16.0 kg/m^2^) persons.

**Table 2:** Person years HRs and associated 95% CIs by sex, categories of BMI and various tobacco habits, MCS, India.

	Hazard Ratios of TB Death					
Tobacco Habits	*BMI (kg/m2) Category*					
	Extremely Thin	Very Thin	Thin	Normal	Overweight	Obese
	(<16.0)	(16.0 to <17.0)	(17.0 to <18.5)	(18.5 to <25.0)	(25.0 to <30.0)	(≥ 30)
**Men**						
Never users						
Person years	3553	3525	9170	85,815	27,238	4407
Observed HR					Reference	
Crude	**30.78(15.82–59.89)**	**10.89(5.00–23.72)**	**4.98(2.35–10.54)**	1.11(0.57–2.16)		-
Adjusted*	**25.40(13.02–49.54)**	**9.34(4.28–20.39)**	**4.54(2.14–9.63)**	1.08(0.55–2.12)		-
Excluding 1st two year deaths†	**15.71(7.45–33.14)**	**8.35(3.59–19.38)**	**2.78 (1.15–6.68)**	0.92(0.45–1.88)		-
Smokeless tobacco users ‡						
Person years	6870	7063	16,471	109,976	29,716	4048
Observed HR						
Crude	**35.29(18.87–66.01)**	**12.03(6.08–23.81)**	**7.91(4.12–15.17)**	**2.17(1.16–4.04)**	0.58(0.23–1.51)	1.86(0.52–6.68)
Adjusted*	**25.50(13.55–48.00)**	**8.93(4.48–17.78)**	**6.04(3.13–11.67)**	1.73(0.92–3.25)	0.49(0.19–1.28)	1.64(0.46–5.87)
Expected HR						
Multiplicative	12.45	4.58	2.22	0.53	0.49	-
Additive	24.89	8.83	4.03	0.57	0.49	-
Excluding 1st two year deaths†	**18.44(9.35–36.38)**	**5.83(2.71–12.58)**	**4.70(2.32–9.55)**	1.30(0.66–2.55)	0.38(0.13–1.12)	1.16(0.25–5.31)
Smoker §						
Person years	7341	6861	14,705	81,727	21,985	3421
Observed HR						
Crude	**43.37(23.36–80.53)**	**15.21(7.79–29.69)**	**9.26(4.83–17.75)**	**2.92(1.56–5.45)**	0.92(0.37–2.29)	1.48(0.33–6.68)
Adjusted*	**36.22(19.41–67.61)**	**13.59(6.93–26.65)**	**8.67(4.51–16.70)**	**2.89(1.54–5.42)**	0.92(0.37–2.30)	1.51(0.34–6.83)
Expected HR						
Multiplicative	23.37	8.59	4.18	0.99	0.92	–
Additive	25.32	9.26	4.46	1.00	0.92	–
Excluding 1st two year deaths†	**22.09(11.25–43.37)**	**10.19(4.90–21.19)**	**5.41(2.63–11.14)**	**2.28(1.17–4.45)**	0.90(0.34–2.36)	1.67(0.37–7.61)
Observed HR*						
*Bidi* #	**41.60(21.98–78.71)**	**16.33(8.15–32.72)**	**9.41(4.74–18.65)**	**3.44(1.78–6.63)**	1.19(0.38–3.77)	–
*Cigarette only*	**22.20(10.42–47.32)**	**7.19(2.65–19.52)**	**7.21(3.26–15.95)**	**2.23(1.13–4.41)**	0.73(0.23–2.31)	1.99(0.44–8.97)
**Women**						
Never users						
Person years	4480	3949	9090	66,646	36,558	12,367
Observed HR					Reference	
Crude	**25.41(8.19–78.79)**	**13.96(3.94–49.48)**	**5.01(1.34–18.65)**	2.33(0.78–6.92)		1.47(0.27–8.07)
Adjusted*	**24.85(7.96–77.56)**	**15.38(4.32–54.72)**	**5.70(1.53–21.32)**	2.42(0.81–7.19)		1.45(0.27–7.95)
Excluding 1st two year deaths†	**26.14(7.02–97.33)**	**13.86(3.09–62.24)**	4.63(0.93–23.03)	1.94(0.53–7.05)		1.93(0.32–11.60)
Smokeless tobacco users ‡						
Person years	13,508	10,122	20,778	102,614	38,268	10,413
Observed HR						
Crude	**29.51(10.57–82.39)**	**12.95(4.26–39.34)**	**7.20(2.41–21.53)**	**3.98(1.43–11.08)**	1.68(0.49–5.75)	0.88(0.10–7.90)
Adjusted*	**19.39(6.79–55.35)**	**9.49(3.06–29.39)**	**5.14(1.68–15.68)**	**2.86(1.01–8.11)**	1.22(0.35–4.22)	0.63(0.07–5.68)
Expected HR						
Multiplicative	30.32	18.76	6.95	2.95	1.22	1.77
Additive	25.07	9.71	5.92	2.64	1.22	1.67
Excluding 1st two year deaths†	**17.58(5.19––59.53)**	**12.14(3.38–43.54)**	**5.71(1.59–20.48)**	2.89(0.87–9.61)	1.38(0.34–5.59)	0.82(0.08–7.94)

Abbreviation: MCS, mumbai cohort study; TB, tuberculosis; HR, hazard ratio; CI, confidence interval; BMI, body mass index  =  weight (kg)/height (m)^2^.

* adjusted for age, education, religion, and mother tongue; note that all hazard ratios significantly different from the referent are denoted by the use of bold font.

† excluding deaths occurring in the first two years to reduce effect of weight loss or smoking cessation due to symptoms of disease.

‡ includes all types of smokeless tobacco products.

§ may include smokers plus mixed (smoking and smokeless) users.

# may include bidi plus cigarette smokers; presented only adjusted HRs.

The observed joint effects of smokeless tobacco use and low-BMI in terms of HRs were lower than the expected HRs (estimated separately by assuming additive and multiplicative interactions, Table 2); indicating antagonistic interaction in women. In contrast, the joint effect of tobacco use in any form (smoking or smokeless) and low-BMI indicated synergistic interaction in men.

Self-reported tuberculosis at the baseline was found to be associated with increased tuberculosis mortality, more in men (HR = 30.30; 95% CI = 24.33–37.79) than in women (HR = 8.99; 95% CI = 2.09–38.78). In contrast, no such association with tuberculosis mortality was observed for both men (HR = 1.15; 95% CI = 0.46–2.89) and women (HR = 2.05; 95% CI = 0.28–15.33) having diabetes.

## Discussion

Worldwide, the effect of two major risk factors underlying the major causes of death, tobacco use and body habitus is now increasing rapidly [9]. MCS had demonstrated that use of tobacco in any form [*6*] and low-BMI [8] independently increased all-cause and cause-specific mortality (e.g., cancer, tuberculosis, etc.). The current study continues these efforts and shows the joint effect of tobacco use and low-BMI on tuberculosis mortality and observed its joint effect being synergistic in men and antagonistic in women.

Individual effect of tobacco use [3]–[6] and BMI [8] on tuberculosis mortality had been reported. In the present study, the marginal HRs ranged from 1.33 (95% CI, 1.07 to 1.66) for being a smokeless tobacco user to 38.21 (25.44 to 57.41) for being extremely thin men and 1.18 (0.80 to 1.71) for being smokeless tobacco user to 17.95 (9.31 to 34.61) for being extremely thin women. Compared to their individual effect the current study clearly highlights the fact that together they increased the risk many fold. This was further strengthened by observing continuous increasing HRs from thin never users to smokers among men (from 4.54 to 36.22) and to smokeless tobacco users among women (from 5.70 to 19.39). The highest such risk was observed among extremely thin male smokers (HR = 36); to be precise among bidi smokers (HR = 41). Risk estimates for bidi smokers appeared to be higher (but not significant) than for cigarette smokers across BMI. This finding strengthened our earlier conclusion [6], [14] that bidi smoking seems to be equally harmful as cigarette smoking [table 2]. Additionally, using HRs from Table 2 we estimated that around 9%, 22%, and 27% of male tuberculosis deaths were attributable respectively to their being either never users, or smokeless tobacco users, or smokers who had BMI <18.5 kg/m^2^. Similarly, these respective proportions were 12% and 37% for female never or smokeless tobacco users who had BMI <18.5 kg/m^2^. Therefore, findings from MCS raise serious concerns about the magnitude of the adverse joint impact of tobacco use and BMI on the health status of the population.

Body weight has been mentioned briefly as a modifier to disease risk in the Centre for Disease Control and Prevention guidelines [19], but little attention has been paid to this readily measurable prognostic index in clinical practice [20]. Low body weight has been associated with risk of tuberculosis disease, severity of disease, unfavorable response to treatment, and relapse [21]. MCS shows association of low-BMI with increased risk of tuberculosis deaths; further, the study reported the risk-increasing trend with increased severity of thinness [*8*]. In contrast the risk-lowering effect of high BMI which was recently observed in elderly patients in Hong Kong [21] was also observed in Mumbai [8]. They were at ∼60% decreased risk of tuberculosis deaths when compared with normal weight persons [*8*]. Although there is evidence of double burden of undernutrition and overnutrition from India [8], [14], [16] public health attention traditionally has focused on problems of undernutrition. The high prevalence of low-BMI [14] and tobacco use [13], [14], [22] and their inter-relation [15] raises alarming concerns about their joint impact on public health in India. This concern is strengthened by findings on joint effect of tobacco use and BMI on all-cause [11] and on cancer [12] and now on tuberculosis mortality in this study.

In MCS, education (proxy for SES) was associated with both tobacco use [13] and BMI [16]. This might have reflected higher tuberculosis mortality among extremely thin persons in Mumbai. One possible explanation might be the relationship between tobacco use, occupation and education [23]. In countries in transition; where less-educated persons are in labor intensive occupations are more likely to be tobacco users, while persons with higher education are living a more sedentary lifestyle.

Subclinical infection with *Mycobacterium tuberculosis* is widespread and smoking seems to facilitate progression from a silent to the active clinical disease, therefore, smoking may contribute to the spread of tuberculosis infection [24]. An association of smoking with tuberculosis was first reported in the 1950s [24], but widespread treatment resulted in tuberculosis becoming too rare to study in high-income countries and therefore largely forgotten. More recently, increased risks of tuberculosis deaths among smokers have been reported in countries where tuberculosis remains common [*2*4]. Using MCS [5] we had reported that smoking accounts for ∼38% of tuberculosis deaths which was further reconfirmed by a nationally representative case-control study [*2*5]. Additionally, MCS finding [5] is consistent with the meta-analysis finding [26] that tuberculosis mortality risk was mostly below the tuberculosis disease risk.

Infectious agents and pollution are the other environment factors that may play a role in this interaction. Tobacco use [27]–[29] and poor nutrition [30] impair the immune system. Hence, tobacco users are more susceptible to infectious agents. On the other hand, infections will increase oxidative stress in tobacco-users. For example, smoking is associated with higher risk of tuberculosis mortality and prevalence of active tuberculosis [5], [6], [24] and with low-BMI [15].

The interactions between malnutrition, tobacco use and infections make persons more vulnerable to smoking-related mortality and morbidity. Besides the direct physiological effect, tobacco use among the economically disadvantaged is known to reduce the resources available to purchase food, clothing, health, and education, all factors that contribute to poor nutritional status [*3*1]. This helps to explain why changes in the relationship between BMI and smoking change with the secular trend toward affluence [*3*2]. So, the current study clearly underscores the importance of joint effect of tobacco use and BMI on public health.

### Strengths and Limitations

The cause of death information obtained from local death registries may be associated with imprecision. On the other hand, the Mumbai Registry is one of the oldest and the most efficient systems of mortality ascertainment and therefore should be most reliable data from this country [33]. Also, we excluded polling stations that served the upper-middle class and upper-class housing complexes because of security issues (i.e. they were essentially “gated communities”). However, it should be noted that they constituted a small proportion during early 1990's. We also excluded persons belonging to the lowest SES, that includes footpath dwellers, because they were not generally included in the electoral rolls and would be very difficult to follow-up [7], [12], [14].

We note that even though we did not have specific data on illness-related weight loss, we had baseline health information on some key categories of major illness for two-third of the cohort. Self-reported tuberculosis at baseline was associated with increased risk of tuberculosis mortality [5]. Therefore, additionally we compared the results after excluding initial deaths that occurred during 1^st^ two years of follow-up to control for potential weight loss owing to pre-existing illness. The overall results remained similar even after exclusion. Deaths that occurred during initial 1^st^ two years of follow-up were mostly of persons aged >60 years (131 out of 289). Considering the average life expectancy in India and the fact that older people tend to lose weight with increasing age, these deaths might be considered as natural deaths. There is some disagreement over the value of excluding initial follow-up (and thus early deaths) to take into account confounding effects owing to pre-existing disease [34]–[36]. So, it is still not conclusive whether or not initial period deaths should be excluded and what should be initial period as its effect may vary from country to country with variance in cause specific distribution. Additionally, based on current study findings stated above, public health researchers might take age of person also into consideration while excluding early deaths. We note that there might be tuberculosis cases younger than 35 years of age in Mumbai who were not available for this analysis because of study eligibility criteria.

Alcohol drinking (HR = 2.53; 95% CI 1.88to3.40) had been reported to increase risk of tuberculosis deaths in Mumbai [7]. We note that alcohol information was available for 35,102 men out of 88,658; consisting in only 192 tuberculosis deaths out of total 710 observed [7]. We had analyzed this subgroup separately; observed HRs (ranged from 1.05 to 29.74) before including alcohol use in the final multivariate model remained similar (ranged from 1.09 to 25.97) after inclusion. Some other possible limitations of this study, including loss to follow-up due to high migration and its impact on tobacco and mortality association [4], [6], [11], [14], limitations in BMI [8], [11], [14], [16] (e.g., very few obese persons and few who have been overweight or obese through most of their adulthood) have been discussed in our earlier publications.

### Conclusions

This study shows that 27% male tuberculosis deaths were attributable to their being underweight and smoker; while 22% male and 37% female tuberculosis deaths attributable to their being underweight and smokeless tobacco user. On the contrary, 9% male and 12% female tuberculosis deaths were attributable to their being underweight and never tobacco user. Tobacco use in any form and low-BMI had joint impact on tuberculosis mortality and the interaction was synergistic in men and antagonistic in women. Considering the fact that currently there are about 275 million tobacco users and about one third of adults being underweight in India; this study underscores the fact that tobacco use and low-BMI may have even more far-reaching public health implications than previously thought. The policy implications for prevention would be that improving the nutritional status of those having low-BMI and preventing use of tobacco in any form may results in the immediate highest yield.
